# Mechanisms of Susceptibility and Resilience to PTSD: Role of Dopamine Metabolism and BDNF Expression in the Hippocampus

**DOI:** 10.3390/ijms232314575

**Published:** 2022-11-23

**Authors:** Vadim E. Tseilikman, Olga B. Tseilikman, Anton A. Pashkov, Irina S. Ivleva, Marina N. Karpenko, Vladislav A. Shatilov, Maxim S. Zhukov, Julia O. Fedotova, Marina V. Kondashevskaya, H. Fred Downey, Eugenia B. Manukhina

**Affiliations:** 1 School of Medical Biology, South Ural State University, 454080 Chelyabinsk, Russia; 2 Department of Basic Medicine, Chelyabinsk State University, 454001 Chelyabinsk, Russia; 3 Federal Neurosurgical Center, 630048 Novosibirsk, Russia; 4 Pavlov Department of Physiology, Institute of Experimental Medicine, 197376 Saint Petersburg, Russia; 5 Laboratory of Neuroendocrinology, Pavlov Institute of Physiology, 199034 Saint Petersburg, Russia; 6 Avtsyn Research Institute of Human Morphology, Petrovsky National Research Center of Surgery, 117418 Moscow, Russia; 7 Department of Physiology and Anatomy, University of North Texas Health Science Center, Fort Worth, TX 76107, USA; 8 Laboratory for Regulatory Mechanisms of Stress and Adaptation, Institute of General Pathology and Pathophysiology, 125315 Moscow, Russia

**Keywords:** hexobarbital sleep test, post-traumatic stress disorder, corticosterone, dopamine, MAO, COMT, BDNF

## Abstract

Susceptibility and resilience to post-traumatic stress disorder (PTSD) are recognized, but their mechanisms are not understood. Here, the hexobarbital sleep test (HST) was used to elucidate mechanisms of PTSD resilience or susceptibility. A HST was performed in rats 30 days prior to further experimentation. Based on the HST, the rats were divided into groups: (1) fast metabolizers (FM; sleep duration < 15 min); (2) slow metabolizers (SM; sleep duration ≥ 15 min). Then the SM and FM groups were subdivided into stressed (10 days predator scent, 15 days rest) and unstressed subgroups. Among stressed animals, only SMs developed experimental PTSD, and had higher plasma corticosterone (CORT) than stressed FMs. Thus, resilience or susceptibility to PTSD was consistent with changes in glucocorticoid metabolism. Stressed SMs had a pronounced decrease in hippocampal dopamine associated with increased expressions of catecholamine-O-methyl-transferase and DA transporter. In stressed SMs, a decrease in monoaminoxidase (MAO) A was associated with increased expressions of hippocampal MAO-A and MAO-B. BDNF gene expression was increased in stressed FMs and decreased in stressed SMs. These results demonstrate relationships between the microsomal oxidation phenotype, CORT concentration, and anxiety, and they help further the understanding of the role of the liver–brain axis during PTSD.

## 1. Introduction

Post-traumatic stress disorder (PTSD) is a long-term consequence of severe acute and/or chronic psychological stress. In addition to behavioral symptoms, such as anxiety, hyperexcitability, anger, and fear, PTSD is often associated with neuroendocrine and metabolic disorders and damage to internal organs [1,2,3,4]. Importantly, among survivors of similar psychological trauma, there are both PTSD-susceptible and PTSD-resilient individuals [5,6].

Previously we found that a hexobarbital sleep test (HST) predicted PTSD resilience in a rat model of experimental PTSD [7,8]. This test characterizes the intensity of microsomal oxidation. Based on the results of the HST, animals were segregated into two phenotypes, fast metabolizers (FM) and slow metabolizers (SM). Even in unstressed rats, the HST test demonstrated a relationship between the intensity of anxiety and these phenotypes of microsomal oxidation. In rats exposed to predator stress (PS), anxiety was elevated only in SMs. In these SMs, activation of the 11βHSD1-dependent pathway of glucocorticoid metabolism was detected. At the same time, SMs had a higher brain activity of monoaminoxidase A (MAO-A), which produces oxidative deamination of monoamine neurotransmitters. Glucocorticoids regulate both MAO-A activity and expression and, thereby, can directly influence the brain metabolism of norepinephrine, DA, and serotonin.

Recently, we identified new factors in the development of experimental PTSD [9]: activation of hepatic 11βHSD1, and reduced plasma corticosterone (CORT) followed by a decrease in brain MAO-A activity, which disturbs the monoamine-neurotransmitter balance. In SMs, PS reduced the cerebral activity of MAO-A.

Low hippocampal dopamine (DA) is associated with an increased risk of PTSD [10,11]. In clinical studies, a so-called “pro-DA regulator,” nutraceutical KB220Z, was successfully used for the treatment of PTSD-related recurrent, distressing nightmares [12]. In our previous PS study [13], cerebral DA concentrations were decreased in PS-exposed rats. In PTSD, the reduced DA concentration is associated with the increased expression of the DA transporter (DAT) and catechol-O-methyltransferase (COMT) genes [14,15]. COMT is an enzyme that, together with MAO-A, is involved in DA metabolism. Earlier we showed that following PS, DA metabolism was impaired while concentrations of DA metabolites were increased [16]. The hippocampus participates in the regulation of anxiety [17,18,19,20,21,22]. The hippocampus interacts with both cortical and subcortical brain regions that regulate emotions and responses to stress. These include the prefrontal cortex, amygdala, hypothalamus, and the adjacent nucleus that regulate the anxiety response to stress [23,24]. Thus, the hippocampus is considered to be particularly vulnerable to chronic stress. Clinical studies of PTSD have revealed a reduction in the hippocampus total volume [25,26,27]. These destructive processes are associated with the disordered expression of brain-derived neurotrophic factor (BDNF), the major neuroplasticity factor [28,29,30,31]. Phenotypic differences between SM and FM are manifested as different concentrations of circulating glucocorticoids [32]. Therefore, it is possible that the SM and FM phenotypes are also different in glucocorticoid-dependent neurobiological and neurochemical parameters in various brain structures, which, in turn, would reflect differences in behavior.

We hypothesized that an increased level of anxiety in SM would be associated with reduced concentrations of BDNF and impaired DA metabolism in the hippocampus. Changes in FM are expected to be either opposite or in the same direction but less pronounced than in SM. This study tested that hypothesis.

## 2. Results

### 2.1. Behavior of SM and FM in Elevated plus Maze

The variables used to compute the anxiety index (AI) values (see equation in Section 4) for each group are detailed in Table 1. AIs of the FMs were significantly lower than those of respective SMs, 10% lower for unstressed rats (*p* < 0.05) and 44% lower for stressed rats (*p* < 0.01). Among FMs, the AI of stressed rats was 25% lower than that of unstressed rats (*p* < 0.01), whereas among SMs, the AI of the stressed rats was 19% greater than that of the unstressed rats (*p* < 0.05).

### 2.2. Effect of PS on Plasma CORT Concentrations in FM and SM Rats 

**Figure 1:**
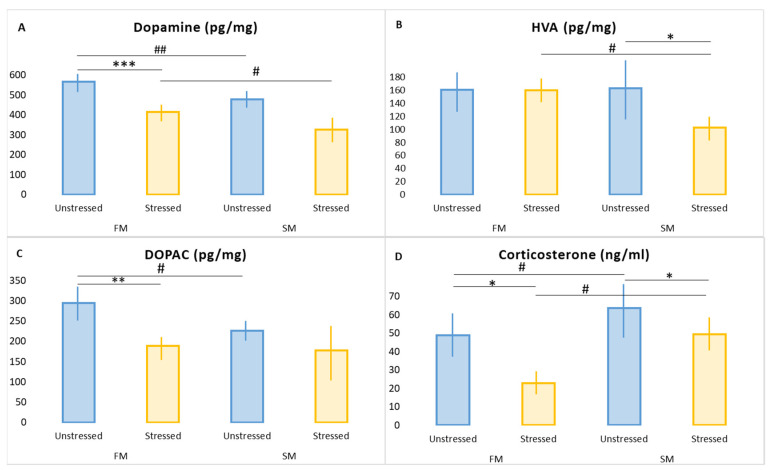
Hippocampal DA (**A**), HVA (**B**) and DOPAC (**C**), and plasma CORT (**D**) in SM and FM rats. SM, slow metabolizer; FM, fast metabolizer; DOPAC, dihydroxyphenylacetic acid; HVA, homovanillic acid. * *p* < 0.05, ** *p* <0.01, *** *p* < 0.001 respective stressed vs. unstressed rats; ^#^ *p* < 0.05, ^##^ *p* < 0.01 respective FM vs. SM.

Plasma CORT concentration was 30% higher in unstressed SMs than in unstressed FMs (*p* = 0.008) (Figure 1). Following the PS completion, the CORT concentration reduced by 54% in FMs (*p* = 0.001) while in SMs, it decreased by 22% (*p* = 0.004) compared to unstressed rats. In unstressed SMs, the CORT concentration was twice as high as in stressed FMs (*p* < 0.001). In the SM group, we found a positive correlation (Supplemental File S1) between corticosterone concentration and anxiety index (r = 0.806; *p* < 0.05).

### 2.3. Effect of PS on Hippocampal DA Metabolism in FM and SM Rats

In unstressed SMs, the hippocampal DA concentration was 16% lower (*p* = 0.004) and the concentration of the DA metabolite, dihydroxyphenylacetic acid (DOPAC), was 14% lower than in unstressed FMs (*p* = 0.006) (Figure 1). There were no significant differences in the concentration of another DA metabolite, homovanillic acid (HVA).

In stressed FM rats, the DA concentration decreased by 19% compared to unstressed rats (*p* = 0.003). The DOPAC concentration decreased by 23% (*p* = 0.0012), whereas the HVA concentration remained unchanged. A positive correlation (Supplemental File S1) between CORT and DOPAC was found (r = 0.8; *p* < 0.05).

In stressed SMs, the DA concentration was 34% lower than in unstressed SMs (*p* = 0.002) and 30% lower than in stressed FMs (*p* = 0.003). The HVA concentration was 34% (*p* = 0.003) lower in stressed SMs than in stressed FM rats, whereas the DOPAC concentration was unchanged.

In stressed SMs, there was a negative correlation (Supplemental File S1) between the hippocampal DA concentration and the AI value (r = −0.555; *p* < 0.05) and between the DA concentration and plasma corticosterone (r = −0.79; *p* < 0.05). We found a negative correlation between the AI and the HVA concentration (r = −0.8; *p* < 0.05) in stressed SMs.

### 2.4. The Effect of PS on Hippocampal DAT, MAO-A, MAO-B, COMT, and BDNF mRNAs in FM and SM Rats

The hippocampal content of MAO-A mRNA increased threefold in stressed SMs compared to unstressed SMs (*p* = 0.002) and fourfold compared to stressed FMs (*p* = 0.001) (Table 2). The content of MAO-B mRNA increased seven times in stressed SMs compared to unstressed SMs (*p* < 0.0001). In stressed SMs, the hippocampal content of MAO-A mRNA positively correlated (Supplementary File S1) with the AI value (r = 0.84; *p* < 0.05) and MAO-B mRNA (r = 0.85 *p* < 0.05). In stressed rats, the hippocampal content of MAO-A mRNA positively correlated with plasma CORT concentration (r = 0.65; *p* < 0.05) and MAO-B mRNA (r = −0.66; *p* < 0.05).

Stressed FM rats showed threefold increases in COMT mRNA (*p* = 0.025) and DAT mRNA (*p* = 0.015) and a twofold increase in BDNF mRNA (*p* = 0.035) compared to unstressed FMs. In contrast, in stressed SMs, the BDNF mRNA content was 43% lower compared to unstressed SMs (*p* = 0.016) and 84% lower compared to stressed FMs (*p* < 0.0001).

Stressed FMs showed negative correlations (Supplementary File S1) between AI and DAT (r = −0.8; *p* < 0.05) and COMT (r = −0.75; *p* < 0.05). Noteworthy, in stressed FMs, the BDNF mRNA content positively correlated with the time spent in the open arms (r = 0.48; *p* < 0.05) and with the number of entries into the open arms (r = 0.39; *p* < 0.05), and negatively correlated with the time spent in the closed arms (r = −0.48; *p* < 0.05) and the AI value (r = −0.8; *p* < 0.05). Stressed FMs showed positive correlations between the contents of DAT mRNA and the BDNF mRNA (r = 0.8; *p* < 0.05) and between the COMT mRNA and the BDNF mRNA (r = 0.82; *p* < 0.05). In addition, a positive correlation was noted between BDNF mRNA level and DA concentration in the hippocampus (r = 0.79; *p* < 0.05).

Stressed SMs showed negative correlations (Supplementary File S2 Table S1) between the BDNF mRNA content, the MAO A mRNA content (r = −0.49; *p* < 0.05) and the MAO-B mRNA content (r = −0.49; *p* < 0.05) with the plasma CORT concentration (r = −0.79; *p* < 0.05). In addition, there was a negative correlation between BDNF mRNA level and AI (r = −0.91; *p* < 0.05) and a positive correlation between BDNF mRNA and DA levels (r = 0.76; *p* < 0.05).

### 2.5. The Effect of PS on Hippocampal and Plasma BDNF Concentrations in FM and SM Rats

**Figure 2:**
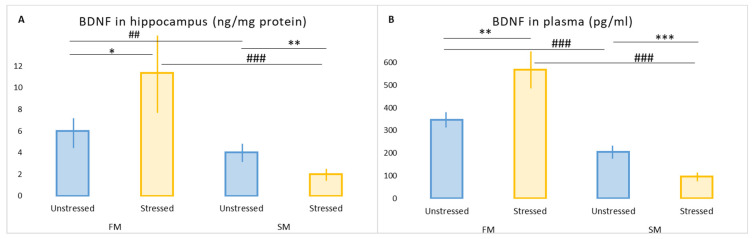
Values are means ± SD. * *p* < 0.05, ** *p* < 0.01, *** *p* < 0.001 vs. respective unstressed rats. ^##^ *p* < 0.01, ^###^ *p* < 0.001 vs. respective fast metabolizers. (**A**) BDNF concentration in hippocampus; (**B**) BDNF concentration in plasma. SM, slow metabolizer; FM, fast metabolizer; DAT, dopamine transporter; MAO, monoaminoxidase; COMT, catechol-O-methyltransferase; BDNF, brain-derived neurotrophic factor.

The hippocampal concentrations of BDNF in stressed FM rats was increased by 90% compared to unstressed FM rats (*p* = 0.032) and by 83% compared to stressed SM rats (*p* = 0.0022) (Figure 2). The plasma concentration of BDNF of stressed FM rats was 60% higher than that of unstressed FM rats (*p* = 0.014) and 83% higher than in stressed SM rats (*p* = 0.0012). In stressed SM rats, the hippocampal concentration of BDNF was 51% lower (*p* = 0.0025), and plasma BDNF was 57% lower (*p* = 0.0014) than in unstressed SM rats. At the same time, in unstressed SM rats, the hippocampal BDNF concentration was 33% (*p* = 0.00012) lower, and the plasma concentration of BDNF was 42% (*p* = 0.00012) lower than in FM rats.

## 3. Discussion

This study showed: (1) In stressed SMs, anxiety correlates with reduced hippocampal concentrations of DA and its metabolites. (2) The decrease in DA is greater in stressed SMs with higher anxiety than in stressed FMs. (3) In FMs, the post-PS decrease in DA is associated with increased expression of COMT and DAT. (4) In stressed SMs, the decrease in MAO-A is associated with increased hippocampal expression of MAO-A and MAO-B mRNAs. In stressed SMs, the decrease in MAO-A is associated with increased hippocampal expression of MAO-A and MAO-B. (5) PS resulted in an increase in the BDNF expression in FMs and a decrease in BDNF expression in SMs. 

Earlier we described the effects of PS on the anxiety of FMs and SMs [8]. Certainly, the ability to identify potential anxiety-like behavior prior to stress requires confirmation with additional behavioral tests, such as open field and light–dark tests, etc. Limited, preliminary confirmation is included in a Supplementary File S2 (Tables S1 and S2). Thus, in most studies, multiple behavioral tests are included in the experimental protocol. However, the animals perceive each behavioral test as a new mild stress [33]. It is possible that each previous test changes the reactivity of the animal, which may significantly affect the results of the next behavioral test. Therefore, we suggested that the administration of two or more tests in a single experimental protocol may not always be relevant, which motivated us for using alternative options. Recently, we have validated tests for the anxiety-like behavior in the same chronic PS model in two experiments [7,8]. In the first experiment, the EPM was used, and in the second experiment, the open-field test was used. In both experiments, similar results were obtained. Meanwhile, we prefer the EPM test because it allows quantification of anxiety-like behavior with the AI, that, in turn, can be used for the segregation of stressed animals into PTSD-susceptible and PTSD-resilient groups. 

Using the same model as in the present study, we observed a decrease in hippocampal DA, that occurred in both high-anxiety and low-anxiety rats. In the present study, the hippocampal concentration of DA decreased in both stressed FMs and SMs. However, this tendency was more pronounced in SMs. Now, we have found that FMs and SMs use different pathways of hippocampal DA reduction.

DA, together with norepinephrine and epinephrine, is metabolized by two monoamine oxidases, MAO-A and MAO-B, and by COMT. DOPAC is a MAO-dependent DA metabolite, whereas HVA is a COMT-dependent metabolite. MAO-A and MAO-B are localized in the mitochondria of presynaptic neurons, and they metabolize DA that returns from the synaptic gap into the presynaptic neuron through DAT. COMT is associated with the postsynaptic neuron and metabolizes DA directly in the postsynaptic gap. Thus, there are two DA metabolic pathways: pathway 1 where the COMT activation predominates and pathway 2 where the MAO activation predominates. During PS, pathway 1 predominated in FMs. This was evident in the increased expression of COMT. Apparently, in stressed FM, DA metabolism on the postsynaptic neuronal membrane was activated. Nevertheless, we cannot exclude that the DA re-uptake was also increased. This possibility is supported by the increased hippocampal expression of DAT. Thus, DA returning to the presynaptic neuron may be repackaged into vesicles and then re-secreted into the synaptic gap. Only secondarily would DA undergo oxidative deamination by MAO-A and MAO-B. In SMs, in contrast, DA oxidative deamination predominates as shown by the increased MAO-A and MAO-B expression. Possibly, this feature of SMs provides a more intensive decrease in DA compared to FMs.

Previously, we reported a decrease in brain MAO-A activity in chronic PS [34]. At first glance, the present results seem to contradict the previous report. However, it should be taken into account that in our previous study, we reported the MAO-A activity while here we report the gene expression. MAO-A is a membrane-associated enzyme that is sensitive to its microenvironment. Increased lipid peroxidation in membranes reduces MAO activity, and chronic PS is associated with increased lipid peroxidation in the brain [35,36,37,38,39].

It is important to note that in stressed SMs, the glucocorticoid concentration was increased compared to stressed FMs. Increased MAO-A expression is known to be glucocorticoid-dependent [40,41,42,43,44,45]. Therefore, we can relate higher glucocorticoid levels with an increased hippocampal expression of MAO-A. In addition, stress-induced, increased glucocorticoid concentrations are associated with reduced hippocampal expression of BDNF [45,46,47]. BDNF exerts various biological effects, such as the prevention of damage and death of neurons and stimulation of neuronal regeneration.

High levels of glucocorticoids are known to disturb the BDNF expression in the hippocampus [46,47]. Reduced CORT would probably cause an opposite effect, i.e., increased BDNF expression. This hypothesis is supported by our previous report that chronic PS resulted in reduced anxiety, decreased plasma CORT, and increased BDNF expression in the hippocampus [48,49,50]. In the present study, the ameliorated anxiety of stressed FMs was associated with an increased hippocampal concentration of BDNF mRNA.

Note that in stressed FMs, the concentration of circulating CORT was lower than in stressed SMs. The BDNF expression was increased in stressed FMs and reduced in stressed SMs. 

Furthermore, in SMs, the decrease in the BDNF gene expression correlated with higher anxiety and the increase in the BDNF gene expression correlated with lower anxiety. In chronic stress, due to the shortage of BDNF, dendrites of hippocampal neurons become shorter, and the neuronal spine density decreases. These changes are considered signs of impaired neuronal plasticity, which adversely affect the hippocampal activity and are associated with both anxiety and depressive behavioral disorders [50,51]. The mechanisms underlying the decrease in BDNF expression in SMs require further study. In this context, the presence of a negative correlation between the hippocampal contents of MAO-A mRNA and BDNF mRNA is noteworthy, since MAO-A inhibitors are known to decrease BDNF expression. It remains unclear to what extent DA metabolic disorders and reduced BDNF expression are interrelated. Are these events independent consequences of the effect of glucocorticoids on hippocampal neurons, or are they interrelated stages of the damage to the hippocampus and the development of behavioral disorders? This must be elucidated in future studies.

This was a pilot study. In the future, the relationship between hippocampal DA and BDNF of stressed FM and SM rats will be investigated. The possibility of such relationships is supported by reports on the trigger role of the BDNF-TrkB signaling pathway in the formation of vesicles with DA [52]. In addition, the “dopamine” hypothesis of the development of anxiety disorders in stressed SMs described here will be tested using the L-DOPA replacement therapy. If this hypothesis is confirmed, it will be possible to enhance the pharmacological correction of PTSD by activation of dopaminergic neurotransmission.

## 4. Materials and Methods

### 4.1. Experimental Animals

The study was performed on 44 male Wistar rats weighing 220–263 g. The rats were kept in standard conditions at a 12:12 h light–dark cycle, with ad libitum access to water and food. All experimental procedures were conducted according to the guidelines of the Declaration of Helsinki and approved by the Animal Care and Use Committee of the Institute of General Pathology and Pathophysiology (Protocol #2, 15 March 2019).

### 4.2. Hexobarbital Sleep Test (HST)

The hexobarbital solution was prepared on the day of the experiment and administered i.p. at a dose of 60 mg/kg 30 days prior to PS. The sleep time after hexobarbital (HST) administration was designated as the time between the injection of hexobarbital and recovery of the righting reflex. The righting reflex was defined as the ability of the animal, after being placed on its back on a flat surface, to turn over by 180° three times within 15 s. Based on the results of the HST test, rats were divided into two groups: FM (sleep duration < 15 min, n = 22, 52% of the study subjects) and SM (sleep duration ≥ 15 min, n = 20, 48% of the study subjects), reflecting the intensity of microsomal oxidation [53].

### 4.3. Predator Stress

The SM and FM groups were subdivided into stressed and unstressed subgroups. The stressed subgroups were exposed to cat urine scent (predator stress, PS) for 10 min daily for 10 days followed by 14 days of rest under stress-free conditions. Unstressed rats rested throughout this period [54].

### 4.4. Behavioral Testing

The behavior of all rats was evaluated with an elevated plus maze (EPM) test using the standard EPM apparatus TS0502-R3 (OpenScience, Moscow, Russia). Recorded variabsles included the time spent in the open and closed arms of the maze and the number of entries into the open and closed arms [55]. The video system SMART with SMART 3.0 software was used. Based on these measurements, an anxiety index [55,56] was calculated: AI = 1 − {[(time in open arms/time on maze) + (number of entries into open arms/number of all entries)]/2}. The EPM test was performed on the 14th day after the end of PS.

### 4.5. Measurement of Plasma Concentration of Corticosterone (CORT)

At 9:00 am, 24 h after the EPM test, rats were decapitated, their blood and tissues collected, and the plasma was separated from the blood cells. Plasma CORT was measured in duplicate with a commercial radioimmunoassay kit (Bioclin, Cardiff, UK) according to the manufacturer’s instructions. The assay sensitivity was 0.25 ng/mL, and the intra- and inter-assay coefficients of variation were both <5%, as previously described.

### 4.6. Measurement of Hippocampal Concentrations of DA and Its Metabolites 

Hippocampal tissue was homogenized in 0.1 M perchloric acid. After homogenization, the samples were centrifuged (7000× *g* for 15 min at 40 °C), and the supernatants were filtered through a syringe Whatman filter with 0.2 µm pores (MilliporeSigma, Burlington, VT, USA) before high-performance liquid chromatography (HPLC). HPLC was performed in the isocratic conditions with electrochemical detection on a Hypersil BDS C18 (250 × 4.6 mm, 5 µm) (Thermo Fisher Scientific, Waltham, MA, USA) reversed-phase column. The mobile phase consisted of a 75 mM phosphate buffer containing 2 mM citric acid, 0.1 mM octanesulphonic acid, and 15% (*v/v*) acetonitrile (pH 4.6).

Electrochemical detection was performed with a glassy carbon electrode at +0780 mV. The final concentration of DA in a tissue sample was expressed as pg/mg tissue using an external calibration curve, as previously described [57,58].

### 4.7. Measurement of mRNAs

#### 4.7.1. RNA Isolation

Total RNA was isolated from the hippocampus using TRIzol Reagent (Invitrogen, Oxford, UK). RNA concentrations were measured using a NanoDrop 2000 spectrophotometer (Thermo Fisher Scientific, Waltham, MA, USA) following the standard procedure. The purity of RNA samples was verified by confirming that each had an optical density ratio A260/A280 > 1.8. To verify the integrity of the samples, the 18S/28S RNA ratio was analyzed after electrophoresis in 1.4% agarose gel.

#### 4.7.2. cDNA Synthesis and Real-Time RT–PCR

Two μg of total RNA was used for cDNA synthesis using high-capacity cDNA reverse transcription kits (Applied Biosystems/Thermo Fisher Scientific, Waltham, MA, USA). Quantitative real-time RT–PCR was performed using Evrogen 5x qPCR mix–HS SYBR (Evrogen, Moscow, Russian Federation). Primers were designed with the Primer-BLAST software (National Centre for Biotechnology Information, Boston, MA, USA) the primer sequences are presented in Table 3. The PCR parameters were as follows: initial denaturation (one cycle at 95 °C for 15 min); 40 cycles of denaturation, amplification, and quantification (95 °C for 15 s, annealing temperature for 30 s, and 72 °C for 5 s); and the melting curve (starting at 65 °C and gradually increasing to 95 °C). Cycr mRNA was used as an internal control. The ΔΔCt method was used to determine the fold increase in genes relative to the control group. The results are given as bar charts. Each value was combined from 2 independent PCR replicates for each cDNA sample, obtained from 5 animals.

Nucleotide sequence of primers used for gene expression measurements. Monoamine oxidase A (MAO-A), monoamine oxidase B (MAO B), catechol-O-methyltransferase (COMT), DA transporter (DAT), brain-derived neurotrophic factor (BDNF).

### 4.8. ELISA Measurements

Hippocampal homogenates and plasma samples were used for the detection of the BDNF, levels by rat ELISA kits (Cusabio Biotech Co., Ltd., Wuhan, China) according to the manufacturer’s instructions. The sensitivity of the rat BDNF ELISA kits was 0.212–20 ng/mL.

### 4.9. Statistical Analysis

Data were analyzed with SPSS 24 (IBM, New York, NY, USA), STATISTICA 10.0 (StatSoft, Tulsa, OK, USA), Rstudio (RStudio, Boston, MA, USA), and Excel (Microsoft, Redmond, WA, USA) software. The normality of data distributions was examined with the Shapiro–Wilk test. Quantitative data were presented as mean ± SD. Two-way ANOVA with Tukey post hoc tests was used to compare all outcome measures between the groups. *p* < 0.05 was considered significant.

## 5. Conclusions

This study showed for the first time biochemical differences in the hippocampus between FM and SM phenotypes of microsomal oxidation in experimental PTSD. In FM rats, reduced anxiety was associated with increased hippocampal expression of BDNF and COMT genes with a simultaneous increase in DAT gene expression. In SM rats, increased anxiety was associated with reduced BDNF gene expression and increased MAO-A and MAO-B gene expression in the hippocampus. The critically important findings included a negative correlation between the plasma concentration of CORT and the hippocampal expression of the BDNF gene and positive correlations between the plasma concentration of CORT and the hippocampal expression of MAO-A and MAO-B genes. These data supplement our previous results showing relationships between the microsomal oxidation phenotype, CORT concentration, and anxiety, and they also further understand the role of the liver-brain axis during PS.

## Figures and Tables

**Table 1:** Effect of PS on behavior of FM and SM rats in EPM.

Variable	FM		SM	
	Unstressed (n = 10)	Stressed (n = 12)	Unstressed (n = 10)	Stressed (n = 10)
Time spent in open arms (sec)	117 ± 18	284 ± 78 **	58 ± 17 ^##^	46 ± 10 ^##^
Time spent in closed arms (sec)	483 ± 18	317 ± 78 **	542 ± 27 ^##^	554 ± 27 ^##^
Entries into open arms	8.2 ± 1.0	8.1 ± 3.9	5.3 ± 1.2 ^##^	2.0 ± 0.5 *^##^
Entries into closed arms	5.7 ± 1.9	4.1 ± 2.7	5.0 ± 3.0	3.3 ± 1.2
AI	0.60 ± 0.04	0.45 ± 0.02 *	0.67 ± 0.08 ^#^	0.80 ± 0.13 *^##^

Values are mean ± SD. * *p* < 0.05, ** *p* < 0.01 vs. respective unstressed rats. ^#^ *p* < 0.05, ^##^ *p* < 0.01 vs. respective fast metabolizers. SM, slow metabolizers; FM, fast metabolizers; AI, anxiety index.

**Table 2:** Gene expression of hippocampal metabolic enzymes and BDNF in FMs and SMs.

	FM		SM	
	Unstressed (n = 10)	Stressed (n = 12)	Unstressed (n = 10)	Stressed (n = 10)
DAT eDAT/GAPDH	0.76 ± 0.09	1.5 ± 0.4 *	2.42 ± 3.24 ^#^	2.54 ± 1.11
MAO-A eMAO-A/GAPDH	0.76 ± 0.3	0.98 ± 0.4	2.39 ± 1.35	6.54 ± 0.97 ***^#^
MAO-B (eMAO-B/GAPDH)	0.61 ± 0.15	2.7 ± 1.36	0.47 ± 0.28	3.28 ± 0.96 *
COMT (eCOMT/GAPDH)	0.96 ± 0.09	1.97 ± 0.35 *	1.04 ± 3.89	2.35 ± 0.83
BDNF eBDNF/GAPDH	1.37 ± 0.49	3.01 ± 0.24 **	0.8 ± 0.2	0.4 ± 0.1 *^###^

Values are means ± SD. * *p* < 0.05, ** *p* < 0.01, *** *p* < 0.001 vs. respective unstressed rats. ^#^ *p* < 0.05, ^###^ *p* < 0.001 vs. respective fast metabolizers. SM, slow metabolizer; FM, fast metabolizer; DAT, dopamine transporter; MAO, monoaminoxidase; COMT, catechol-O-methyltransferase; BDNF, brain-derived neurotrophic factor.

**Table 3:** The primer sequences.

Name of the Gene	Primer Sequence 5′ → 3′	Annealing Temperature, °C
MAO-A	F GCCAGGAACGGAAATTTGTA R TCTCAGGTGGAAGCTCTGGT	64
MAO-B	F TGGGCCAAGAGATTCCCAGTGATG R AGAGTGTGGCAATCTGCTTTGTAG	60
Comt	F CTGGAGGCCATCGACACCTA R AGTAAGCTCCCAGCTCCAGCA	60
BDNF	F GAAAGTCCCGGTATCAAAAG R CGCCAGCCAATTCTCTTTTTG	60
DAT	F TTGGGTTTGGAGTGCTGATTGC R AGAAGACGACGAAGCCAGAGG	55

## Data Availability

Not applicable.

## Supplementary Materials

The following supporting information can be downloaded at: https://www.mdpi.com/article/10.3390/ijms232314575/s1.
