# Supplementary material for: Mechanisms of Susceptibility and Resilience to PTSD: Role of Dopamine Metabolism and BDNF Expression in the Hippocampus
Source: Int J Mol Sci. 2022 Nov 23;23(23):14575. doi: 10.3390/ijms232314575 (PMC9737079; doi:10.3390/ijms232314575)
Supplement: Supplementary file 1 [file ijms-23-14575-s001.zip › ijms-1972162-supplementary.pdf]

Supplemental Files

Supplemental File S1

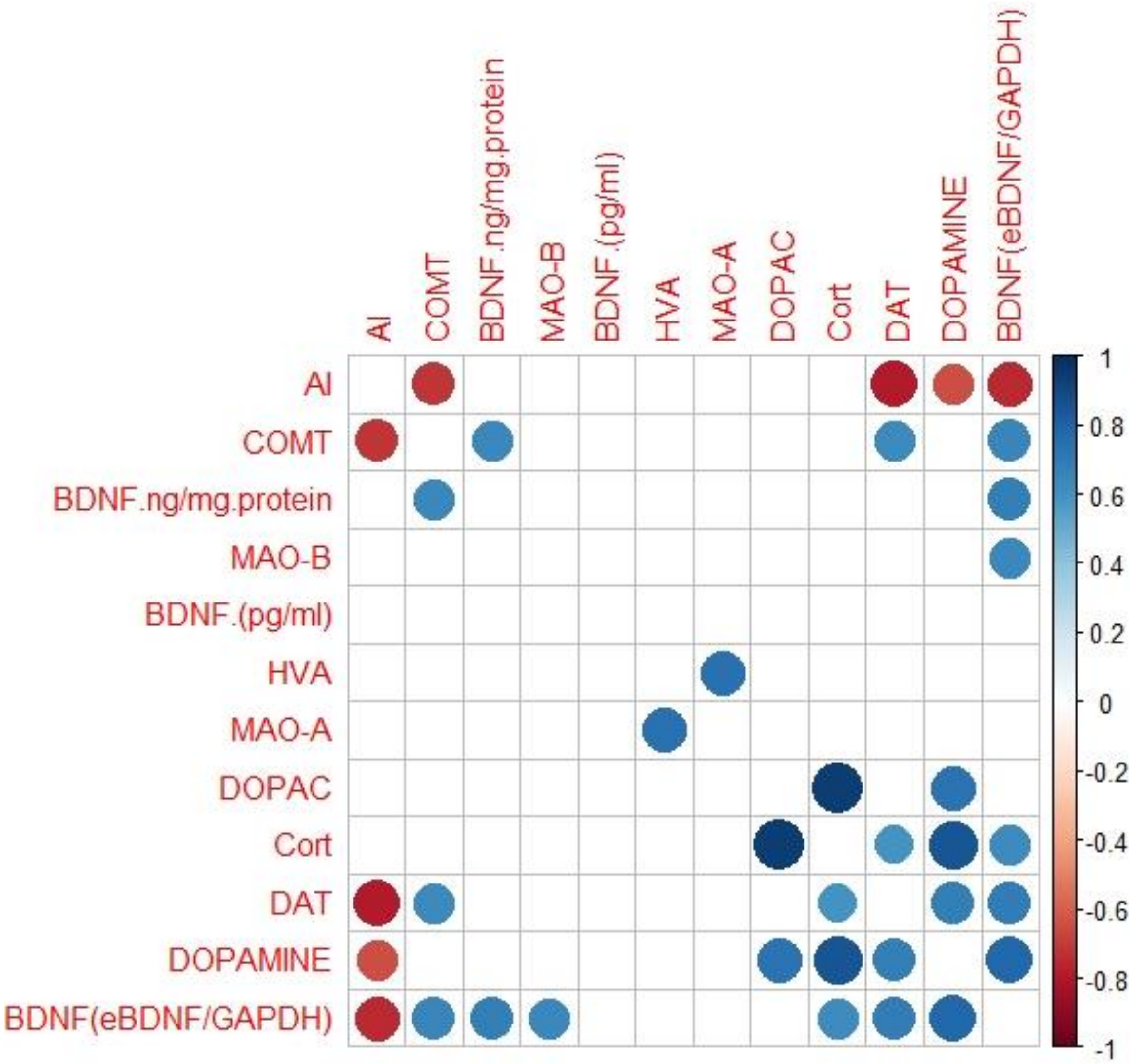

Correlation matrix of biomarkers and anxiety index (AI) in the group of fast metabolizers (FM).

All colored dots depicted in the matrix represent statistically significant ($p<0.05$) correlation coefficients between the tested variables, with the dot size and color intensity (see scale on the right) epresenting the relative magnitude of the correlation. Red: negative correlations; blue: positive correlations.

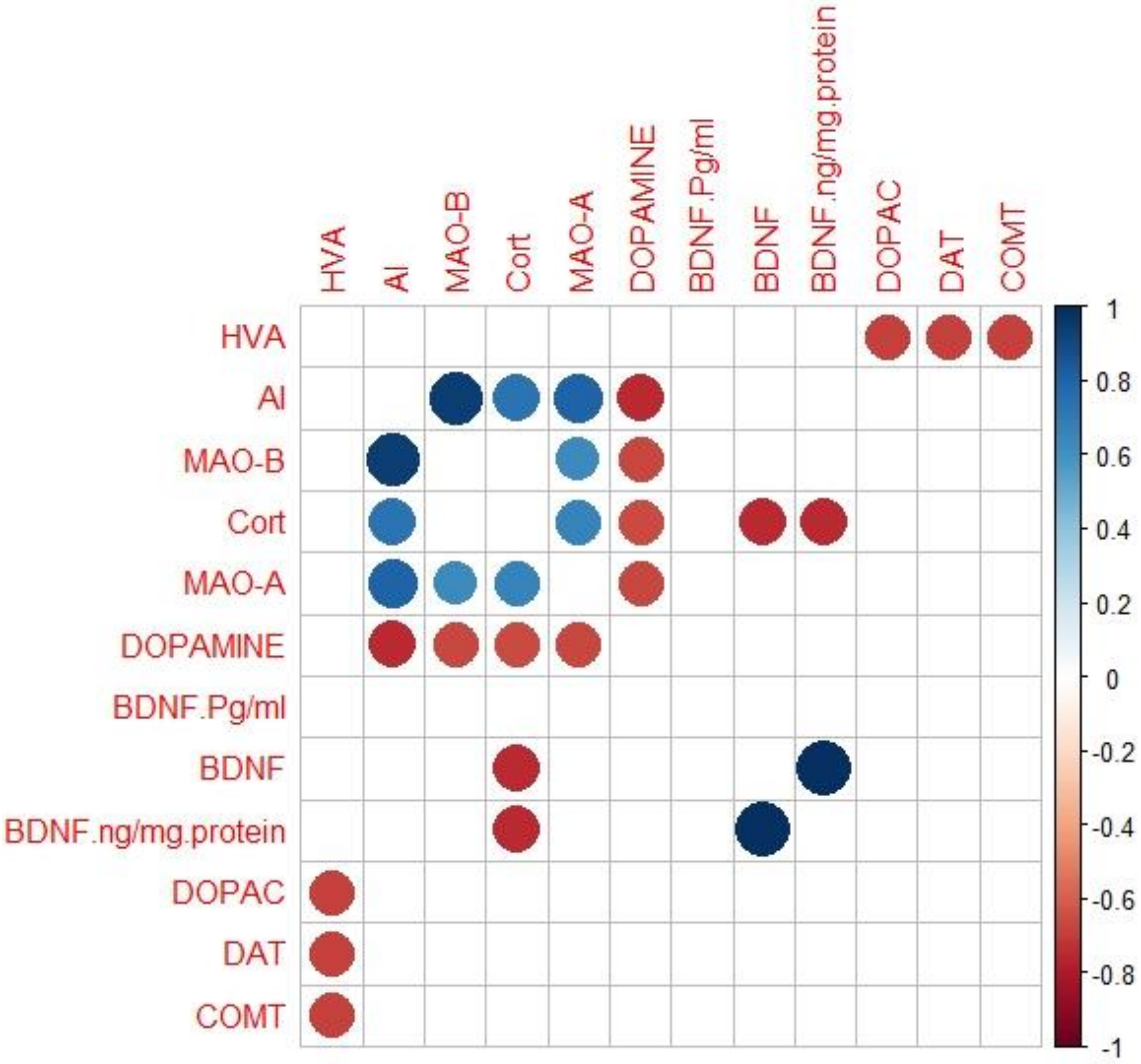

Correlation matrix of biomarkers and anxiety index (AI) in the group of slow metabolizers (SM).

All colored dots depicted in the matrix represent statistically significant ($p<0.05$) correlation coefficients between the tested variables with the dot size and color intensity (scale on the right) representing the relative magnitude of the correlation. Red: negative correlations; blue: positive correlations.

Supplemental File S2

Table S1. Behavior of fast (FM) and slow metabolizers (SM) in the open field test 20 days prior to predator stress

	SM (n=23)	FM (n=33)
Number of crossed sectors/10 min	59.7±6.95	81.4±6.45**
Vertical activity, number of rearings/10 min	2.4±1.37	5.5±0.67**
Freezing, number of acts/10 min	3.85±0.34	1.05±0.14**
Grooming, number of acts/10 min	5.90±0.29	3.60±0.17**
anxiogenic defecation numbers of boluses	4.2± 0.48	1.69± 0.15**

In this experiment, the level of anxiety was assessed at 10 days after the hexobarbital sleep test and 20 days before predator stress. Values are mean±SD. **p <0.01.

Table S2. Behavior of fast (FM) and slow metabolizers (SM) in the elevated X-maze at 14 days after the end of predator stress

Variable	FM		SM	
	Unstressed (n=16)	Stressed (n=17)	Unstressed (n=11)	Stressed (n=12)
Time spent in open arms (sec)	127±15	176.5±23**	84±11$^{\#\#}$	53±9*$^{\#\#\#\#}$
Time spent in closed arms (sec)	473±25	423.5±14**	516±85$^{\#\#}$	547±63*$^{\#\#\#\#}$
Entries into open arms	5.5±0.1	9.3±0.4**	3.4±0.07$^{\#}$	4±0.5
Entries into closed arms	6.5±0.7	3.3±0.9**	8.7±0.24	14.7±1.24
AI	0.67±0.2	0.59±0.4*	0.72±0.1	0.84±0.5$^{\#}$

FMs and SMs were tested on day 14 after the end of predator stress. The results indicated increased anxiety of stressed SMs evident as more frequent freezing and defecation acts as well as reduced

locomotor activity. Values are mean±SD. *$p < 0.05$, **$p < 0.01$ *vs*. respective unstressed rats. $^{\#}p < 0.05$, $^{\#\#}p < 0.01$, $^{\#\#\#}p < 0.001$ vs respective fast metabolizers.
